# Covered stents versus Bare-metal stents in chronic atherosclerotic Gastrointestinal Ischemia (CoBaGI): study protocol for a randomized controlled trial

**DOI:** 10.1186/s13063-019-3609-8

**Published:** 2019-08-20

**Authors:** Louisa J. D. van Dijk, Jihan Harki, Desirée van Noord, Hence J. M. Verhagen, Jeroen J. Kolkman, Robert H. Geelkerken, Marco J. Bruno, Adriaan Moelker, on behalf of the Dutch Mesenteric Ischemia Study group (DMIS), Ron Balm, Gert Jan de Borst, Juliette T. Blauw, Marco J. Bruno, Olaf J. Bakker, Louisa J. D. van Dijk, Hessel C. J. L. Buscher, Bram Fioole, Robert H. Geelkerken, Jaap F. Hamming, Jihan Harki, Daniel A. F. van den Heuvel, Eline S. van Hattum, Jan Willem Hinnen, Jeroen J. Kolkman, Maarten J. van der Laan, Kaatje Lenaerts, Adriaan Moelker, Desirée van Noord, Maikel P. Peppelenbosch, André S. van Petersen, Pepijn Rijnja, Peter J. van der Schaar, Luke G. Terlouw, Hence J. M. Verhagen, Jean Paul P. M. de Vries, Dammis Vroegindeweij

**Affiliations:** 1 Department of Gastroenterology and Hepatology, Erasmus MC University Medical Center, ’s-Gravendijkwal 230, 3015 CE Rotterdam, The Netherlands; 2 Department of Radiology, Erasmus MC University Medical Center, ’s-Gravendijkwal 230, 3015 CE Rotterdam, The Netherlands; 3 Department of Gastroenterology and Hepatology, Franciscus Gasthuis and Vlietland, Kleiweg 500, 3045 PM Rotterdam, The Netherlands; 4 Department of Vascular Surgery, Erasmus MC University Medical Center, ’s-Gravendijkwal 230, 3015 CE Rotterdam, The Netherlands; 5 Department of Gastroenterology and Hepatology, Medisch Spectrum Twente, Postbus 50 000, 7500 KA Enschede, The Netherlands; 6 Department of Gastroenterology and Hepatology, University Medical Center Groningen, Postbus 30.001, 9700 RB Groningen, The Netherlands; 7 Department of Vascular Surgery, Medisch Spectrum Twente, Postbus 50 000, 7500 KA Enschede, The Netherlands; 8 TechMed Centre, Faculty Science and Technology, University Twente, Postbus 50 000, 7500 KA Enschede, The Netherlands

**Keywords:** Endovascular revascularization, Bare-metal stent, Covered stent, Chronic mesenteric ischemia, Atherosclerosis, Celiac artery, Superior mesenteric artery

## Abstract

**Background:**

Chronic mesenteric ischemia (CMI) is the result of insufficient blood supply to the gastrointestinal tract and is caused by atherosclerotic stenosis of one or more mesenteric arteries in > 90% of cases. Revascularization therapy is indicated in patients with a diagnosis of atherosclerotic CMI to relieve symptoms and to prevent acute-on-chronic mesenteric ischemia, which is associated with high morbidity and mortality. Endovascular therapy has rapidly evolved and has replaced surgery as the first choice of treatment in CMI. Bare-metal stents (BMS) are standard care currently, although retrospective studies suggested significantly higher patency rates for covered stents (CS). The Covered stents versus Bare-metal stents in chronic atherosclerotic Gastrointestinal Ischemia (CoBaGI) trial is designed to prospectively assess the patency of CS versus BMS in patients with atherosclerotic CMI.

**Methods/design:**

The CoBaGI trial is a randomized controlled, parallel-group, patient- and investigator-blinded, superiority, multicenter trial conducted in six centers of the Dutch Mesenteric Ischemia Study group (DMIS). Eighty-four patients with a consensus diagnosis of atherosclerotic CMI are 1:1 randomized to either a balloon-expandable BMS (Palmaz Blue with rapid-exchange delivery system, Cordis Corporation, Bridgewater, NJ, USA) or a balloon-expandable CS (Advanta V12 over-the-wire, Atrium Maquet Getinge Group, Hudson, NH, USA). The primary endpoint is the primary stent-patency rate at 24 months assessed with CT angiography. Secondary endpoints are primary stent patency at 6 and 12 months and secondary patency rates, freedom from restenosis, freedom from symptom recurrence, freedom from re-intervention, quality of life according the EQ-5D-5 L and SF-36 and cost-effectiveness at 6, 12 and 24 months.

**Discussion:**

The CoBaGI trial is designed to assess the patency rates of CS versus BMS in patients treated for CMI caused by atherosclerotic mesenteric stenosis. Furthermore, the CoBaGI trial should provide insights in the quality of life of these patients before and after stenting and its cost-effectiveness. The CoBaGI trial is the first randomized controlled trial performed in CMI caused by atherosclerotic mesenteric artery stenosis.

**Trial registration:**

ClinicalTrials.gov, ID: NCT02428582. Registered on 29 April 2015.

**Electronic supplementary material:**

The online version of this article (10.1186/s13063-019-3609-8) contains supplementary material, which is available to authorized users.

## Background

Chronic mesenteric ischemia (CMI) is the result of insufficient blood supply to the gastrointestinal (GI) tract and is caused by atherosclerotic stenosis of one or more mesenteric arteries in > 90% of cases [1, 2]. The mesenteric arteries are the celiac artery (CA), superior mesenteric artery (SMA) and inferior mesenteric artery (IMA). Classic symptoms of CMI are postprandial abdominal pain and weight loss due to fear of eating. However, CMI may present atypically with constant abdominal discomfort, nausea, vomiting, diarrhea or even constipation [1]. The “classic triad” of CMI consisting of postprandial abdominal pain, weight loss and abdominal bruit is present in only 16–22% of patients [3, 4].

The diagnosis of CMI is established by consensus in a multidisciplinary meeting joined by vascular surgeons, gastroenterologists and interventional radiologists [1]. Consensus is an accepted method of diagnosing if a gold standard test is absent [5]. Symptoms alone do not predict the diagnosis of CMI accurately [4, 6, 7]. Therefore, consensus diagnosis is based on the combination of clinical symptoms, radiological evaluation of the mesenteric vasculature and, if available, assessment of mucosal ischemia with a functional test such as gastric-jejunal tonometry [3, 8, 9] or visible light spectroscopy (VLS) [10, 11]. A definitive diagnosis of CMI is established if successful therapy of patients with CMI consensus diagnosis results in a durable relief of presenting symptoms.

Revascularization therapy is indicated in patients with a diagnosis of atherosclerotic CMI to relieve symptoms and to prevent acute-on-chronic mesenteric ischemia, which is associated with high morbidity and mortality. Endovascular revascularization has largely replaced open surgical revascularization [1, 12, 13]. Endovascular revascularization of the mesenteric arteries is achieved by means of stent placement. The SMA and CA are target vessels for therapy because of their larger diameter compared to the IMA. Furthermore, a protective collateral network ensures blood supply when the IMA is occluded as shown in patients with an aortic stent occluding the IMA. Literature on revascularization of the IMA is scarce. A study reported successful stenting of the IMA in four patients who were not candidates for CA or SMA revascularization [14]. The classic percutaneous approach for mesenteric endovascular procedure is trans-brachial and trans-femoral, but a trans-radial approach is currently gaining popularity [15].

Endovascular revascularization is associated with a significant decreased risk of in-hospital complications compared to open mesenteric surgical revascularization [16]. Despite the advantages of endovascular revascularization short term, restenosis is a common event occurring in 28–55% of patients within 2 years after endovascular mesenteric stenting [13, 17] whereas 0–25% of surgically treated patients develop restenosis [2, 18]. The primary patency rate of open surgical revascularization is significantly higher than that of endovascular revascularization (cumulative odds ratio (OR) 3.57, 95% confidence interval (CI) 1.82–6.87, *p* = 0.0002, [19].

Currently, balloon-expendable bare-metal stents (BMS) are standard care for mesenteric endovascular revascularization [1, 20]. Retrospective data, however, showed significantly higher primary patency rates of balloon-expendable covered stents (CS) at 3 years for mesenteric artery stenosis [21]. Furthermore, patients treated with CS had less restenosis, symptom recurrence and re-intervention than patients treated with BMS [21]. The Covered versus Balloon Expandable Stent Trial (COBEST) showed significantly higher patency rates of CS than BMS for aortoiliac arterial disease 18, 24, 48 and 60 months after stent placement [22, 23]. Possibly, the membrane that covers the vascular atherosclerotic lesion functions as a physical barrier for intimal hyperplasia and is, therefore, associated with less restenosis in contrast with BMS. The performance of CS for mesenteric artery stenosis is promising, but prospective confirmation is lacking. The CoBaGI trial is a multicenter, randomized controlled, patient- and investigator-blinded clinical trial designed to compare patency rates of the CS versus standard care therapy with BMS in patients with CMI based on atherosclerosis.

### Study hypothesis

CS have a significantly higher patency rate than BMS in patients with CMI due to stenosis of the CA and/or SMA of atherosclerotic origin.

## Methods/design

This study protocol is written according to the Standard Protocol Items: Recommendations for Interventional Trials (SPIRIT) 2013 Statement for study protocols of clinical trials [24]. The SPIRIT Diagram is shown as Table 1 and the SPIRIT Checklist is added in Additional file 1.

**Table 1:** Schedule of enrollment, interventions and assessments for the CoBaGI trial according to Standard Protocol Items: Recommendations for Interventional Trials (SPIRIT) [24]

	Study period						
	Enrollment	Allocation	Post allocation				Close-out
Time point	−t_1_	0	t_1_	t_2_	t_3_	t_4_	t_x_
Enrollment:							
Eligibility screen	X						
Informed consent	X						
Allocation		X					
Interventions:							
Intervention CS			X				
Intervention BMS			X				
Assessments:							
CTA	X			X	X	X	
EQ-5D-5 L	X			X	X	X	
SF-36	X			X	X	X	
Cost-effectiveness	X			X	X	X	
SAE				X	X	X	X
Mortality				X	X	X	X

−t_1_ = screening and enrollment, t_1 =_ endovascular-stent placement, t_2_ = 6 months, t_3_ = 12 months, t_4_ = 24 months after intervention, t_x_ = end of follow-up at 24 months

*BMS* bare-metal stent, *CS* covered stent, *CTA* computed tomography angiography, *SAE* serious adverse event, *SF-36* 36-item Short Form Health Survey

### Study design

The CoBaGI trial is a randomized controlled, parallel-group, patient- and investigator-blinded, superiority, multicenter trial conducted in six centers of the Dutch Mesenteric Ischemia Study group (DMIS). Patients with atherosclerotic CMI will be randomly allocated to standard care therapy using a BMS (Palmaz Blue with rapid-exchange delivery system, Cordis Corporation, Bridgewater, NJ, USA) or a CS (Advanta V12 over-the-wire, Atrium Maquet Getinge Group, Hudson, NH, USA).

### Study setting

The CoBaGI trial is conducted within the framework of the DMIS. Six Dutch centers participate in the CoBaGI trial. The initiating center is Erasmus MC University Medical Center, an academic hospital in Rotterdam together with Medisch Spectrum Twente in Enschede which serves as a CMI-specialized referral center. Furthermore, four other Dutch tertiary referral centers participate in the study: Maasstad Hospital in Rotterdam; St. Antonius Hospital in Nieuwegein; Bernhoven Hospital in Uden, and Jeroen Bosch Hospital in ‘s-Hertogenbosch.

### Eligibility criteria

Patients with a consensus diagnosis of atherosclerotic CMI eligible for endovascular-stent placement will be included in this trial.

Inclusion criteria are:

- A consensus diagnosis of CMI based on atherosclerotic mesenteric artery stenosis at the origin of the CA and/or SMA, established in a multidisciplinary meeting attended by a gastroenterologist, interventional radiologist and vascular surgeon. This consensus diagnosis is based on:
  - Typical symptoms (presence of postprandial pain, unexplained weight loss)
  - Significant stenosis of > 50% of at least one of the mesenteric arteries on previous computed tomography angiography (CTA)*
  - Functional assessment of mucosal ischemia with VLS or tonometry
- Aged ≥18 years old
- Signed informed consent
- Total length of mesenteric stenosis < 25 mm

*Requirements for CTA are: CTA performed within the year before consensus diagnosis  maximum slice thickness 1 mm, enhancement in aorta 300HU.

Exclusion criteria are:

- Absence of informed consent
- Aged < 18 years old
- No stenosis detected during angiography
- Renal insufficiency (glomerular filtration rate (GFR) below 30 ml/min or GFR below 60 ml/min in the presence of comorbidities relevant to renal function)
- Previous stent placement in the target vessel
- Pregnancy
- Stenosis based on median arcuate ligament syndrome (MALS)
- Stenosis based on vasculitis
- Other criteria which the physician considers incompatible with inclusion in this trial

### Recruitment and consent

Patients with CMI based on atherosclerotic mesenteric artery stenosis are eligible for the CoBaGI trial. A gastroenterologist, surgeon or research physician will screen the patient according to the inclusion and exclusion criteria. When the patient is eligible for study inclusion, the treating physician will inform the patient about the possibility of participating in the CoBaGI trial and will hand out the patient information form (Additional file 2). Furthermore, the treating physician will inform the patient that study participation is voluntary and that participation may be withdrawn at any time without the need of having to provide a reason. At the return visit, the patient is given the opportunity to ask questions. If the patient consents to participate in the CoBaGI trial, written informed consent is obtained.

### Randomization

Patients are 1:1 randomized for a BMS or CS with a web-based randomization module (9 Knots Business Solutions Ltd., Mansfield, UK) in random blocks of four or six stratified by center.

### Interventions

**Fig. 1:**
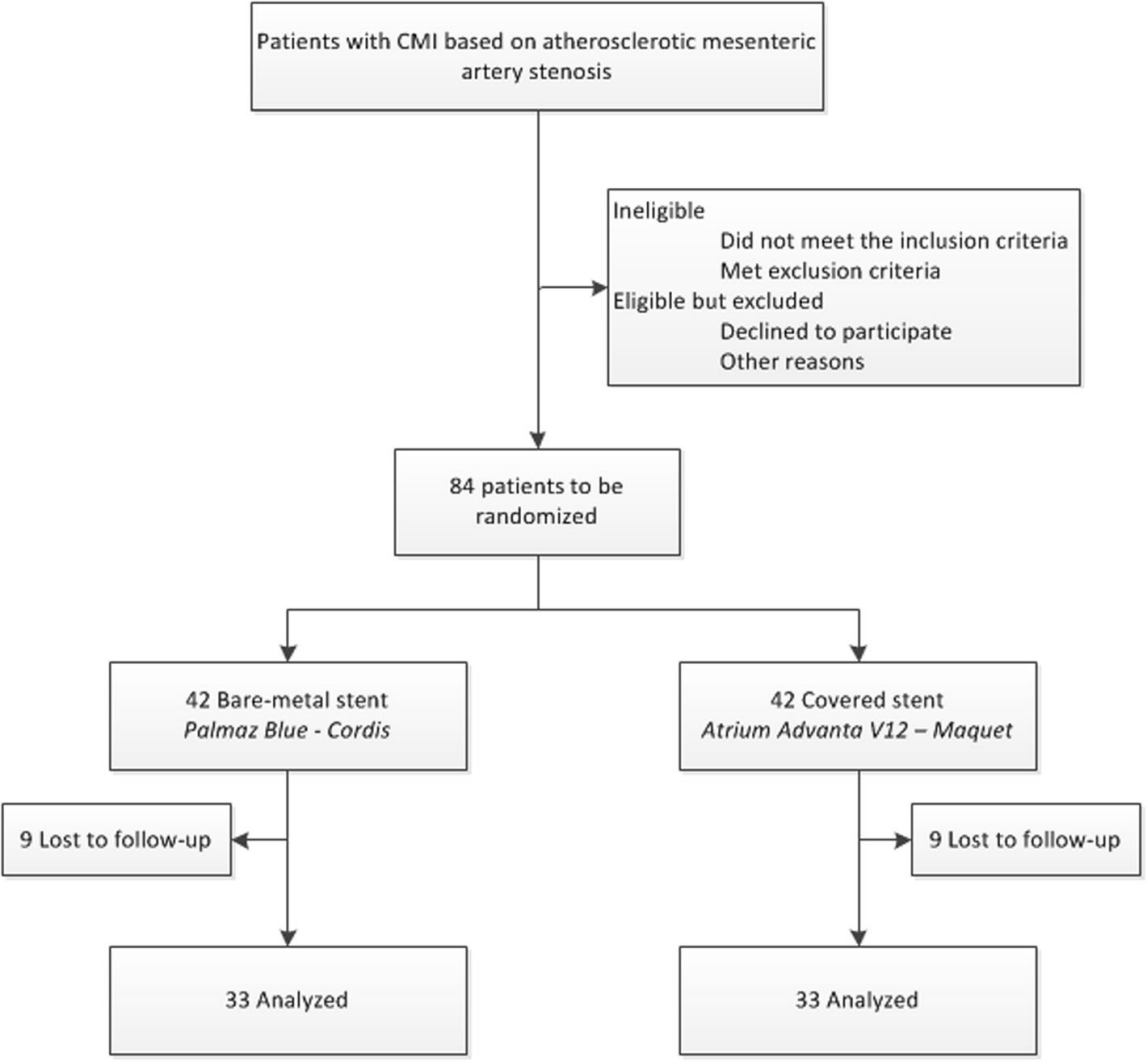
Flowchart of participants in the Covered stents versus Bare-metal stents in chronic atherosclerotic Gastrointestinal Ischemia (CoBaGI) trial according to Consolidated Standards of Reporting Trials (CONSORT) [25]

At the start of the endovascular procedure patients are randomized for either a BMS or CS. The approach of the intervention is at the discretion of the attending interventionalist (femoral, brachial, radial or retrograde). During the angiography the stenoses are assessed and if a suspected stenosis is not significant, this vessel will not be treated. If the stenosis is detected to be significant during angiography and the stenosis length is < 25 mm the stenosis will be treated with the allocated stent. In case multiple stenosis are present and eligible for inclusion (i.e., CA and SMA), the same allocated stent type is used. After completion of the intervention various procedural details, such as duration, approach, stent length, treated vessels and complications, are entered in web-based case record forms by the interventionalist. The antiplatelet regimen in the CoBaGI trial consists of aspirin and clopidogrel combined for 12 months followed by aspirin lifelong and applies for both treatment arms. Table 1 presents the schedule of enrollment, interventions and assessments for the CoBaGI trial and Fig. 1 presents the Consolidated Standards of Reporting Trials (CONSORT) study flowchart [25].

### Follow-up

After the endovascular procedure, the patients are followed up at 6, 12 and 24 months with CTA to assess stent patency and with a visit to the outpatient clinic to assess symptoms and bodyweight. The patients also receive questionnaires to assess quality of life and cost-effectiveness before stent placement and 6, 12 and 24 months after stent placement.

### Outcome

The primary endpoint is the primary patency rate 24 months after stent placement based on intention-to-treat analyses.

The secondary endpoints are (definitions are shown in Table 2):

- Primary patency rates at 6 and 12 months
- Secondary patency rates at 6, 12 and 24 months
- Freedom from restenosis at 6, 12 and 24 months
- Freedom from symptom recurrence at 6, 12 and 24 months
- Freedom from re-intervention at 6, 12 and 24 months
- Quality of life at 6, 12 and 24 months
- Cost-effectiveness at 6, 12 and 24 months

**Table 2:** Definitions

	Definition
Restenosis	> 50% intra-stent stenosis regardless whether the patient had clinical symptoms
Symptom recurrence	Occurrence of presenting symptoms regardless of stent patency
Re-intervention	Intervention due to symptom occurrence in the presence of > 50% intra-stent stenosis, either a reimplantation of stent, or a surgical procedure

### Blinding

The patient, investigator and the treating physician at the outpatient clinic are blinded for the treatment allocation (BMS or CS). For the duration of the trial the allocated stent will not be disclosed unless there is a medical need (i.e., restenosis, stent fracture).

### Sample size

The sample size calculation for this randomized controlled, superiority trial is based on the retrospective data of Oderich et al. [21]. Assuming that CS improves the patency rate of BMS after 24 months from 63% to 95%, and accounting for a 20% dropout rate, 42 participants are required per arm to achieve 80% power with a two-sided alpha of 0.05. The sample size calculation is performed using the Comparison of Independent Proportions Test (SAS Power and Sample size, SAS Institute Inc., Cary, NC, USA).

### Data collection methods

Clinical data are collected using web-based case record forms (9 Knots Business Solutions Ltd., Mansfield, UK). Quality of life is assessed by the validated EuroQol five-dimension, five-level questionnaire (EQ-5D-5 L) [26, 27] and 36-item Short-Form Health Survey (SF-36) [28, 29] questionnaires. The cost-effectiveness questionnaire deals with work omission, hospital visits and use of healthcare. All trial documents are kept for 15 years after study completion.

### Data management

Data entry is performed by local study personnel or personnel of the initiating study center. Data entry is anonymized replacing all patient names with a code. Only these codes are used as reference in reports and publications about this investigation. The web-based case record forms contain range checks for data values. The handling of personal data is compliant with the Dutch Personal Data Protection Act, the Code of Good Behavior and the General Data Protection Regulation effective since May 2018.

### Data monitoring

Institution of a data monitoring committee (DMC) was waived by the Ethical Committee because the added risk of the interventional study arm is considered to be negligible. An interim analysis will not be performed. The initiating center, Erasmus MC University Medical Center, is responsible for the data monitoring activities to ensure data quality.

### Statistical methods

Descriptive analyses will be provided regarding patient characteristics before randomization. Because this is a randomized controlled trial, statistical tests to detect differences in these baseline characteristics between both intervention arms will not be performed. The following patient characteristics before randomization will be provided: age, gender, Body Mass Index (BMI), smoking status, comorbidity with hypertension, dyslipidemia, diabetes and cardiovascular disease. Furthermore presenting symptoms will be described as abdominal pain, postprandial pain, exercise-related pain, nausea, diarrhea, weight loss and symptom duration. Patient characteristics will be described as numbers and percentages for dichotomous variables, or as means and standard deviations for continuous variables with normal distribution or medians and interquartile ranges (IQRs) for continuous variables if not normally distributed.

The primary analysis will be performed according the intention-to-treat principle. A per-protocol analysis will also be performed for the primary outcome of stent patency for those patients who were treated according to the treatment protocol.

The primary endpoint of stent patency will be analyzed with a “two-sample z-test” for comparison of two proportions. Furthermore, a predictive model for time-to-event will be obtained by proportional hazards regression and Kaplan-Meier analysis to compare patency rates of CS versus BMS. All other continuous outcome measures will be reported as difference in mean improvement between the CS versus the BMS group. Change in quality of life assessed by the validated questionnaires EQ-5D-5 L [26, 27] and SF-36 [28, 29] will be compared between the CS and BMS groups. Furthermore, the costs of both groups will be compared. All costs will be estimated based on actual input in terms of resource use, personnel and indirect costs from loss of productivity assessed by the cost-effectiveness questionnaire.

### Harms

Adverse events are defined as any undesirable experience occurring to a subject during the study, whether or not considered related to the implantation of a BMS or CS. A serious adverse event (SAE) is any untoward medical occurrence or effect that at any dose:

- Results in death
- Is life threatening (at the time of the event)
- Requires hospitalization or prolongation of existing inpatients’ hospitalization
- Results in persistent or significant disability or incapacity
- Is a congenital anomaly or birth defect
- Any other important medical event that may not result in death, be life threatening or require hospitalization, may be considered a serious adverse experience when, based upon appropriate medical judgment, the event may jeopardize the subject and may require an intervention to prevent one of the outcomes listed above

All physicians who are involved in the trial are asked to report all adverse events to the coordinating investigator. The coordinating investigator will report the SAEs through the web portal ToetsingOnline (https://www.toetsingonline.nl) of the Dutch Central Committee on Research involving human subjects to the accredited Institutional Review Board that approved the protocol, within 15 days after the coordinating investigator has first knowledge of the serious adverse reactions. Serious adverse events that result in death or are life threatening should be reported expedited. The expedited reporting will occur not later than 7 days after the responsible investigator has first knowledge of the adverse reaction. This is for a preliminary report with another 8 days for completion of the report.

### Auditing

Representatives of the initiating center, Erasmus MC University Medical Center, audit the participating centers during the course of the study. All protocol modifications are communicated to the relevant parties.

### Research ethics approval

The CoBaGI trial is performed in accordance with declaration of Helsinki and the Dutch law regarding research involving human subjects (Wet Medisch wetenschappelijk Onderzoek met mensen (WMO)). The Institutional Review Board of Erasmus MC University Medical Center, Rotterdam, the Netherlands approved the study protocol on 15 October 2013. The boards of the other five Dutch participating centers gave permission for conducting the trial in their center. The CoBaGI trial was registered on 29 April, 2015 at ClinicalTrials.gov with identification number NCT02428582.

### Protocol amendments

Five protocol amendments were approved after initial approval of the CoBaGI protocol by the Institutional Review Board. The subjects of these amendments were the addition of new inclusion centers, the addition of one inclusion criterion and adjustment of the patient information form. The contents of these amendments have been incorporated into this protocol.

### Confidentially

The investigators and the study staff will keep all information about the study patients during and after the trial in strict confidence. All study data are saved in the web-based case record forms. This study database is anonymized, the names of the enrolled study patients are replaced with a patient study number.

### Ancillary and post-trial care

Compensation for post-trial care, for those patients who suffered harm from trial participation, is covered by trial health insurance.

### Dissemination policy

The study protocol and study results will be presented at scientific conferences and in peer-reviewed publications. Authorship eligibility follows the common standards of author responsibility, conflict of interest, transparency and the recommendations of the International Committee of Medical Journal Editors (http://www.icmje.org). There are no limitations or restrictions for publication.

## Discussion

The CoBaGI trial is designed to compare the patency rate of BMS (standard care) with CS in patients with CMI based on atherosclerotic mesenteric stenosis. Besides, the study should provide insights into the change in quality of life of patients induced by both stents and their cost-effectiveness. The CoBaGI trial is the first randomized controlled trial to compare BMS with CS in patients with CMI based on an atherosclerotic mesenteric stenosis.

The recently published clinical practice guidelines of the European Society of Vascular Surgery (ESVS) recommend with a low level of evidence (C: expert opinion and/or small studies, retrospective studies) to consider CS over BMS for patients requiring mesenteric artery stenting based a single retrospective study by Oderich et al. [21]. A stronger, level-A recommendation can only be issued based on the results of randomized controlled trials. The optimal design would be a double-blinded controlled trial. Since the physician who performs the endovascular procedure cannot be blinded, the CoBaGI trial is designed as a patient- and investigator-blinded, randomized controlled trial.

The primary endpoint of the CoBaGI trial is primary stent patency assessed with CTA. The primary endpoint is a hard endpoint which can be assessed objectively. The secondary endpoint of symptom recurrence is dependent on reports of patient and investigator. In order to ensure unbiased collection and an objective assessment of the endpoints, blinding of the patient and investigator is important.

To perform a fair comparison between the two stent designs all participating centers will use the same brand/type of BMS as standard of care in the CoBaGI trial. The initiating center, Erasmus MC University Medical Center, had already ample experience with the BMS Palmaz Blue from Cordis for mesenteric stenting and, therefore, this stent was chosen to be used in all participating centers. BMS from different manufacturers are considered comparable in use and outcome although studies comparing BMS from different manufacturers are lacking.

Of note is the inclusion criterion that the length of the stenosis cannot exceed 24 mm because all stenoses needing stent extension had to be excluded in the CoBaGI trial since a CS will obstruct blood flow when a side-branch is over-stented in contrast to a BMS. The longest available Palmaz Blue BMS during the inclusion of the CoBaGI trial had a length of 24 mm and the longest CS had a length of 59 mm.

A pivotal inclusion criterion for the CoBaGI trial is a consensus diagnosis of CMI based on atherosclerotic mesenteric artery stenosis which is established in a multidisciplinary meeting joined by gastroenterologists, interventional radiologists and vascular surgeons. Medisch Spectrum Twente and Erasmus MC University Medical Center are specialized CMI referral centers. All patients eligible for the CoBaGI trial, therefore, will be discussed during a CMI multidisciplinary meeting in one of these two centers. If functional assessment of mucosal ischemia is required to reach a consensus diagnosis, especially in case of single-vessel disease, the patient is referred to Erasmus MC University Medical Center (VLS) [10, 11] or Medisch Spectrum Twente (24-h tonometry) [3, 8, 9].

After endovascular revascularization for CMI, antiplatelet therapy is recommended and dual antiplatelet therapy might be considered for 3–12 months [1]. Exact dose and duration schedules are lacking for mesenteric revascularization. All stented patients in the CoBaGI trial, regardless of allocation, will receive aspirin and clopidogrel for 12 months after stenting followed by aspirin lifelong.

The true incidence of atherosclerotic CMI is unknown. It is expected that in the upcoming years the incidence of atherosclerotic CMI will increase, mainly because of the aging population and the increasing prevalence of cardiovascular disease. The predicted increase in the prevalence of atherosclerotic CMI, the significant patient burden in terms of pain and loss of quality of life, and, in particular, the risk of an acute ischemic event with an exceedingly high mortality risk, underscore the importance of the CoBaGI trial. The CoBaGI trial aimed to improve the patient outcome in terms of pain relief, improvement of quality of life and prevention of acute ischemic events. The fact that the CoBaGI trial is carried out within the framework of the DMIS with patients being recruited from multiple academic and community hospitals should facilitate the generalizability of the study outcomes.

In conclusion, the CoBaGI trial is a multicenter, randomized controlled, patient- and investigator-blinded superiority trial comparing BMS (standard of care) with CS in patients with CMI caused by an atherosclerotic stenosis at the origin of the mesenteric artery.

### Trial status

The CoBaGI trial started inclusion in May 2015. In September 2018, 71 patients of the 84 (85%) patients had been included. Inclusion is expected to be completed at the end of 2018. Follow-up duration is 2 years. Latest protocol version is version 5, date 28 June 2017.

## Additional files

Additional file 1: Standard Protocol Items: Recommendations for Interventional Trials (SPIRIT) 2013 Checklist: recommended items to address in a clinical trial protocol and related documents*. (DOC 121 kb)

Additional file 2: Translated patient information form of the CoBaGI trial. (DOCX 46 kb)

## Data Availability

The datasets used and/or analyzed during the current study are available from the corresponding author on reasonable request.

## Abbreviations

BMI: Body Mass Index
BMS: Bare-metal stent(s)
CA: Celiac artery
CMI: Chronic mesenteric ischemia
CS: Covered stents
CTA: Computed tomography angiography
DMC: Data Monitoring Committee
DMIS: Dutch Mesenteric Ischemia Study group
GFR: Glomerular filtration rate
GI: Gastrointestinal
IMA: Inferior mesenteric artery
IQR: Interquartile range
MALS: Median arcuate ligament syndrome
SAE: Serious adverse event
SMA: Superior mesenteric artery
VLS: Visible light spectroscopy
WMO: Wet Medisch wetenschappelijk Onderzoek met mensen (Dutch law regarding research involving human subjects)
