# Supplementary material for: Covered stents versus Bare-metal stents in chronic atherosclerotic Gastrointestinal Ischemia (CoBaGI): study protocol for a randomized controlled trial
Source: Trials. 2019 Aug 20;20:519. doi: 10.1186/s13063-019-3609-8 (PMC6700968; doi:10.1186/s13063-019-3609-8)
Supplement: Supplementary file 2 — Translated patient information form of the CoBaGI trial. (DOCX 46 kb) [file 13063_2019_3609_MOESM2_ESM.docx]

**Patient information folder**

**Study title**

The comparison between metal and covered stents in the treatment of chronic gastrointestinal ischemia.

**Introduction**

**Dear Sir / Madam**

You have been referred to the Erasmus MC for examination and treatment of your gastrointestinal complaints. Investigations have shown that you have a circulatory problem of the stomach and small intestine caused by narrowing or stenosis of one or more abdominal arteries due to arteriosclerosis. This is called chronic gastrointestinal ischemia. The treatment of chronic gastrointestinal ischemia consists of placing a tube, also called a stent, in the stenotic abdominal artery.

You are asked to participate in a scientific study, since you are eligible for treatment of your circulatory problem of the stomach and small intestine by placing a stent in the stenotic abdominal artery. The study is called 'The comparison between metal and covered stents in the treatment of chronic gastrointestinal ischemia'. We kindly ask you to participate in this medical-scientific study.

Before you make the decision to participate or not, it is important to know more about the study. This information folder explains the content of the study and the value of this study next to the standard investigations. Read this information folder carefully. Discuss it with your partner, friends or family. Good information is required on our part for permission or refusal to participate and a careful consideration on your part. At the end of this information folder you will find a declaration of consent in which you can indicate whether you wish to participate.

Do you still have questions after reading the information folder? Then you can ask your doctor at the outpatient clinic of the department of Gastroenterology and Hepatology of the Erasmus MC or by contacting the researchers. There is also an independent physician, who is well informed about the study. You will find the contact details at the end of this information folder. Also read the General Brochure for Medical-Scientific Research, which contains general information about medical-scientific research.

**1. What is the purpose of this study?**

With this study we want to investigate which stent is better for treatment of a circulatory problem of the stomach and small intestine. We perform this study with patients with a stenosis of one or more abdominal arteries who are eligible for treatment with a stent in the stenotic abdominal artery. Stent placement will restore the blood supply to the stomach and small intestine.

An important complication of stent placement is re-stenosis of the stent or in-stent stenosis, mainly caused by the inflammatory reaction resulting from damage to the blood vessel when the stent is placed. In-stent stenosis can lead to reduced blood flow to the stomach and small intestine. The symptoms which were present before the stent was placed can return when in-stent stenosis occurs. If in-stent stenosis is symptomatic, treatment is required. To treat in-stent stenosis, a new stent has to be placed in the blood vessel. This means hospital admission again for the patient, associated with extra costs.

In the Erasmus MC, the standard treatment of chronic gastrointestinal ischemia consists of placing a metal stent in the stenotic blood vessel. However, new stents have been developed recently called covered stents. Covered stents are also made of metal, but are covered with an artificial layer, whereby the chance of in-stent stenosis is decreased since the damage of the blood vessel is reduced during stent placement.

The goal of this study is to investigate which type of stent is better for the treatment of chronic gastrointestinal ischemia: bare-metal stents or covered stents. We want to investigate which type of stent leads to decreased complications, such as the in-stent stenosis and the recurrence of symptoms. Therefore, you will visit the outpatient clinic for two years after stent placement.

**2. Which treatment is being investigated?**

The standard treatment for a circulatory problem of the stomach and small intestine consists of placing a metal stent. We want to investigate which stent is responsible for less in-stent stenosis after treatment since in-stent stenosis leads to recurrence of symptoms and re-treatment. With this study we investigate the treatment of a gastrointestinal circulatory problem of the stomach and small intestine by placing a metal stent or a covered stent.

**3. How is the study conducted?**

You will undergo stent placement in the stenotic abdominal artery to restore blood and oxygen supply to the stomach and small intestine. The standard treatment consists of placing a metal stent in the stenotic abdominal artery by an interventional radiologist. The standard treatment is performed as follow:

After locally anesthetization, an artery in your wrist, upper arm or grown will be punctured. A thin tube (catheter) will be inserted through the artery, which will be guided to the abdominal vessels. As soon as it is past the stenosis, contrast fluid will be applied through the catheter in the arm to make the blood vessels and stenosis more visible with the help of X-rays (angiography). If you have had ever a (serious) allergic reaction during a previous examination with iodinated contrast agent, we ask you to inform your doctor in advance so that any precautionary measures can be taken. The metal stent will be inserted through the tube to the belly vessel and revolves stenosis. This is the standard treatment. If you decide not to participate in the study, you will undergo the standard treatment.

If you decide to participate in the study, the procedure will be as described above. Instead of a metal stent, a covered stent will be placed in the stenotic abdominal artery.

In order to compare two types of stents well, it is important that the groups are comparable. This means that as many people get a metal stent as a covered stent. In order to ensure that there is no difference between these groups, so that the chance of success of the treatment cannot be influenced, the allocation to the type of stent will be determined by drawing lots. This means that you cannot predetermine the type of stent, nor the doctor can determine that for you. Your attending physician at the outpatient clinic also does not know which stent you are receiving or have received. The allocated stent will be announced at the end of the study. You can read more about the preparation and execution of the treatment in the General Information Folder on Angiography.

If you decide to participate in this study, you will visit the outpatient clinic during 24 months after stent placement with visits at 6, 12 and 24 months. During a visit, we will ask you about possible (returned) symptoms. We will also perform imaging of your abdominal vessels using a CT scan to assess whether the vessel with the stent has not become stenotic. To get good images of your abdominal vessels, it is necessary to administer an iodinated contrast agent intravenously. The infusion needle is inserted into the preparation room or into the examination room. The infusion needle is removed approximately 15 minutes after the CT scan.

You can read more about the preparation and execution of the CT scan in the General Information Folder on CT scan.

With a short questionnaire, we will also ask you how you judge your quality of life in terms of your health and general functioning on a scale from 0 to 100, where 0 is the worst possible outcome and 100 the best score imaginable. To make a correct comparison, this is done once for the treatment and 6, 12 and 24 months after the treatment.

In appendix 1 Study procedures you will find a flow chart with an overview of everything that will be done during the entire study.

In appendix 2 Questionnaire you will find the short questionnaire that you must complete before and after the treatment.

**4. What is expected from you?**

We expect no extra effort from you. The preparation for this study is the same as for the standard treatment. If you decide to participate in this study, we ask you to keep the agreements as well as possible, to follow the instructions as well as possible and to keep the doctor informed of all changes in your health condition that you feel, during and after you have undergone the stent placement.

If you decide to participate in the study, you will remain under our control for 24 months. This is the same period as if you decide not to participate in the study.

**5. What is more or different than the regular treatment(s) you receive?**

If you do not wish to participate in this study, you will receive the standard treatment. The standard treatment consists of placing a metal stent. After the treatment you will visit the outpatient clinic 6, 12 and 24 months after stent placement. Your possible symptoms will be evaluated and you will be examined physically.

If you participate in this study, there is a chance that you will get a covered stent instead of a metal stent to eliminate the stenosis in your abdominal artery. Which stent you will receive will be determined by drawing lots. Even then, you will visit the outpatient clinic 6, 12 and 24 months after stent placement. In addition, to evaluate your symptoms and physical examination, a CT scan of your abdominal vessels will be made. Furthermore, you will complete a short questionnaire about your health and general functioning once before the stent placement and three times after the treatment.

In appendix 1 Study procedures you will find a flow chart with an overview of everything that will be done during the entire study compared to the standard treatment.

**6. What are the other possible treatments?**

If you do not participate in this study, you will receive the standard treatment for a circulatory problem of the stomach and small intestine. This consists of placing a metal stent in the stenotic abdominal artery.

**7. What are possible side effects and risks that you can expect?**

Treatment with a covered stent instead of a metal stent is not associated with extra risks. The procedure stent placement is the same. Furthermore, no extra visits to the outpatient clinic are planned if you participate in this study. However, when you participate in this study you have to undergo a CT scan in order to assess your abdominal vessels at 6, 12 and 24 months after treatment.

During the CT scan, iodinated contrast agent is administered intravenously to assess the blood vessels and any stenosis. In the vast majority of cases, the administration of contrast agent proceeds without problems. The infusion needle is removed approximately 15 minutes after the Ct scan. If you have ever received a (serious) allergic reaction during a previous investigation with iodinated contrast agent, please inform us and your doctor in advance so that precautionary measures can be taken.

In a small number of patients an allergic reaction to the contrast agent occurs, which usually consists of sneezing or the formation of hives. In most cases, this requires no further treatment. A serious allergic reaction is extremely rare. The team that carries out the study is trained in preventing and treating such problems.

If you are familiar with a kidney condition, please inform your doctor. We will also check repeatedly whether your kidneys are functioning properly. In the event that your kidneys function less well, it may be necessary to administer fluid before and after the CT scan intravenously.

The study is performed with as little x-rays as necessary for good quality imaging. The X-rays you will receive during the entire study are negligible. However, it is important to inform your doctor immediately if you are pregnant or you could be pregnant. In consultation with the radiologist, the investigation can be continued or postponed, or an alternative investigation can be performed possibly.

Because we would also like to know how you experience your health and general functioning, we ask you to fill in a short questionnaire just once before stent placement and three times after stent placement.

**8. What are possible advantages and possible disadvantages of participating in this study?**

If you participate in this study, you will receive a CT scan at set times to assess your abdominal arteries and the inserted stent. A stenosis of the abdominal artery due to in-stent stenosis can be detected earlier.

Disadvantage of extra CT scan is the increase of X-ray radiation. In order not to expose you to more radiation than is necessary, the X-ray radiation will be limited to your abdomen. The dose of X-rays in a CT scan is small and comparable with the natural background radiation in the Netherlands for 1.5 years. The CT scan duration is 30 minutes per scan.

During this study you have to complete the questionnaire about your health and general functioning for four times. Expected duration of completing the questionnaire is 5 minutes.

In total, the entire study will therefore take about 2 hours longer than the usual treatment with participating in the study.

**9. What happens if you do not wish to participate in this study?**

You decide whether you will participate in this study or not. Participation in this study is entirely voluntary. If you do not want to participate, you do not have to give a reason for this. If you decide not to participate, this will not change your further treatment or guidance, you will receive the standard treatment for your symptoms. A metal stent is placed for standard treatment and you will visit the outpatient clinic just as often after the treatment compared to participating in the trial. If you decide not to participate, only one CT scan will be performed and no questionnaires will have to be completed. If you give permission to participate in this study, you can always withdraw from this study without giving any reason.

**10. What happens when the study is finished?**

You can stop participating in this study whenever you wish. You do not have to give a reason why you want to stop participating. The researcher can stop your participation in this study if he / she feels that participation is no longer desirable for you (for example because you experience too many side effects).

At the end of the study you can - if you wish - get an overview of the results. We would like to point out that these results are described at group level. You will not receive any personal results from the study. Any findings that are of interest to you personally will be discussed with you by the researcher.

**11. Are you insured when you take part in this study?**

An insurance policy is taken out for everyone who participates in this study. The insurance covers damage as a result of this study. This applies to damage that comes up during the study, or within four years after the end of the study.

You can find more information about this insurance in Appendix 3 Insurance.

**12. Are you informed about personally relevant information about the study during the study?**

The study will be performed as accurate as possible. But the situation can change. For example, by the reaction of your body, or by new information. If so, we will discuss this directly with you. If your safety or well-being is in danger, we stop the study immediately

**13. What happens with your data?**

The general brochure explains that the researcher collects data about you and treats it confidentially. This means that a number of persons may view your medical status and the data of the study. These persons may use the data for this study, but they may only disclose this information without mentioning your name or other personal details. Your identity is always secret. The researcher saves the data with a code. This means that the study documents only contain a letter number code instead of your name. Only the researcher keeps a list that states which letter-number code belongs to which name.

Normally, only your attending physician and his / her team have access to your data. If you participate in this study, more people will see your medical data and study data. The persons who can view your data are:

- the staff of the study team,

- the members of the review committee who approved the study,

- the authorized employees of the Health Care Inspectorate

- the monitor that monitors the study

- the safety committee that monitors the study.

After the study, the coded data is stored for 15 years. This is necessary to be able to check everything properly. In addition, we would like to use your data for other studies that are performed on gastrointestinal ischemia. These studies therefore have the same goal as the study for which you are now being asked. It is therefore not the case that your data will also be used for studies into a completely different condition or a completely different problem. Naturally, the confidentiality that we described above always applies. Do you agree if we save and use your data? If you do not want that, we naturally respect that. You can indicate your choice on the consent form.

**14. Is your general practitioner and / or attending specialist informed when participating?**

We will inform your doctor that you are participating in the study. This is for your own safety. You must give permission for this on the consent form. If you do not give permission, you cannot participate in the study.

**15. Are there extra costs / is there a compensation if you decide to participate in this study?**

You will not receive any compensation for participating in this study.

**16. Which medical ethics review committee approved this study?**

The Medical Ethical Assessment Committee Erasmus MC has received approval for this study.

**17. Would you like to know more?**

If you have questions or complaints during the study, we ask you to contact the researcher or your doctor.

- Principal investigator Dr. A. Moelker (Interventional radiologist, department of Radiology) at telephone number 010-7042006.

- L.G. Terlouw (research-physician, department of Gastroenterology and Hepatology) on telephone number 06-34951247.

If you have doubts about participation, you can consult an independent doctor, who is not involved in the study yourself, but who is competent in the field of this study and your illness.

Even if you have questions before or during the study that you would rather not ask the researchers, you can contact the independent physician.

- Dr. A.C. Weustink (Radiologist, department of Radiology) 06 - 14795616

If you are not satisfied with the investigation or the treatment, you can contact the independent complaints committee of the Erasmus MC. They can be reached at telephone number: 010-7033198

Good information is required on our part for permission or refusal to participate in this study and a careful consideration on your part. Read this information folder carefully. If you decide to participate in this scientific study after careful consideration, we ask you to sign and date the consent form together with the researcher.

Sincerely,

The study team

**18. Appendices**

Appendix 1 Consent form

Appendix 2 Study procedures

Appendix 3 Insurance

Appendix 4 Questionnaires

General Information Folder Medical-Scientific Research with People

General information brochure Angiography

General information folder CT scan

**Appendix 1 consent form**

The comparison between metal and covered stents in the treatment of chronic gastrointestinal ischemia.

I have read the information folder. I could ask additional questions. My questions have been answered. I had enough time to decide whether to participate or not in this study.

I know that participating is completely voluntary. I know that at any moment I can decide not to participate anyway. I do not have to give a reason for that.

I do / do not * give consent to tell my doctor that I am participating in this study.

I do / do not * give consent to tell the specialist treating me that I am participating in this study.

I know that some people can see my data. These people are listed in the General brochure and in this information folder.

I give permission to use my data for the purposes stated in the information folder.

I give permission to keep my study data for 15 years after the end of this study.

I do / do not * give consent to be approached again in the future for further studies.

I want to participate in this study.

Naam of subject:

Signature: Date : __ / __ / __

-----------------------------------------------------------------------------------------------------------------

I hereby declare that I have fully informed this subject about the study mentioned.

If during the study relevant information becomes known that could influence the consent of the study subject, I will notify him or her rapidly.

Name researcher (or his representative):

Signature: Date: __ / __ / __

-----------------------------------------------------------------------------------------------------------------

* Delete what is not applicable.

**Appendix 2 Study procedures**

**Treatment of blood circulation problems of the stomach and small intestine:**

| Treatment of blood circulation problems of the stomach and small intestine | Standard treatment | Study participation |
| --- | --- | --- |
| Questionnaire | **NO** | YES |
| Stent placement | YES | YES |
| Visits at outpatient clinic at 6, 12 and 24 months after stent placement | YES | YES  +  CT-scan |

**Study procedures:**

| **Start study** | - Complete questionnaire about health and general functioning  - Stent placement in stenotic abdominal artery |
| --- | --- |
| **Time = 6 months** | - Visit outpatient clinic + CT scan of the abdominal vessels  - Complete questionnaire about health and general functioning |
| **Time = 12 months** | - Visit outpatient clinic + CT scan of the abdominal vessels  - Complete questionnaire about health and general functioning |
| **Time = 24 months** | - Visit outpatient clinic + CT scan of the abdominal vessels  - Complete questionnaire about health and general functioning |

**Appendix 3: Insurance**

An insurance policy is taken out for everyone who participates in this study. The insurance covers damage resulting from participation in the study. This applies to damage that comes up during the study, or within four years after the end of the study. You must have reported the damage to the insurer within 4 years.

In the event of damage, you can contact the insurer directly.

The insurer of the study is:

Meeus Assurantien BV

Mrs. N.Munnix

PO Box 234

6040 AE Roermond

The insurance offers a maximum coverage of € 450,000 per subject, with a maximum amount of € 3,500,000 for the entire study. If the client of this study has conducted several investigations, a maximum amount of € 5,000,000 per policy year applies for all investigations. The coverage of specific damages and costs is further limited to certain amounts. This can be found in the compulsory insurance policy for medical research with people. Information about this can be found on the website of the Central Committee on Human Research: www.ccmo.nl.

A number of exclusions also apply to this insurance. The insurance does not cover:

- damage of which (on the basis of the nature of the investigation) it was (almost) certain that it would occur;

- damage to health that would also have arisen if you had not participated in the study;

- damage resulting from (incomplete) compliance with instructions or instructions;

- damage to offspring, as a result of an adverse effect of the examination on you or your offspring;

- in research into existing treatment methods: damage resulting from one of these treatment methods;

- in research into the treatment of specific health problems: damage resulting from lack of improvement or worsening of these health problems.
